# Hippo–YAP/TAZ signaling in inner ear development, injury, and regeneration: evidence synthesis and the biomechanical brake hypothesis

**DOI:** 10.3389/fcell.2026.1878367

**Published:** 2026-09-23

**Authors:** Dongmei Guan, Xinghong Liu, Xiaoyi Zhang, Jingqi Zhang, Hui Xie

**Affiliations:** 1 College of Clinical Medicine, Chengdu University of Traditional Chinese Medicine, Chengdu, China; 2 Affiliated Hospital of Chengdu University of Traditional Chinese Medicine, Chengdu, China

**Keywords:** biomechanical brake, cochlear supporting cells, hair-cell regeneration, Hippo–YAP/TAZ signaling, inner ear regeneration, mechanotransduction, organ of corti

## Abstract

Sensorineural hearing loss in mammals is essentially irreversible. Mature cochlear hair cells, once lost, do not regenerate. Non-mammalian vertebrates retain this ability, but in the mammalian organ of Corti, supporting cells lose their proliferative and transdifferentiation competence after birth. Reactivation of classical developmental pathways has also shown limited success in the adult cochlea. In this review, we first synthesize published evidence on Hippo–Yes-associated protein(YAP)/transcriptional coactivator with PDZ-binding motif (TAZ) signaling in the inner ear, including studies of inner-ear progenitors, organ of Corti growth, vestibular sensory epithelia, cochlear hair-cell injury, neonatal cochlear regeneration models, and emerging adult cochlear data. On this basis, we then evaluate the biomechanical brake hypothesis, which proposes that injury-associated mechanical remodeling may interact with Hippo–YAP/TAZ signaling to restrict supporting-cell plasticity in the mature mammalian cochlea. Rather than presenting this hypothesis as an established mechanism, we treat it as an evidence-graded framework that must be tested against alternative explanations, including protective epithelial repair, cell-cycle inhibition, epigenetic restriction, and loss of developmental competence. The available literature suggests that Hippo–YAP/TAZ signaling participates in several inner-ear processes, including progenitor expansion, sensory epithelial growth, species-dependent vestibular repair, otoprotective responses, and supporting-cell proliferation under defined experimental conditions. However, direct evidence that injury-induced mechanical remodeling causally suppresses YAP/TAZ-dependent regeneration in the adult cochlea remains limited. We therefore distinguish among developmental, neonatal, vestibular, *ex vivo*, and adult *in vivo* evidence, and identify where current findings support, weaken, or remain insufficient to establish the biomechanical brake model. Finally, we outline specific gaps that should guide future work: comprehensive tabulation of Hippo-related inner-ear studies, adult supporting-cell lineage tracing, spatial mapping of cochlear mechanical properties and Hippo activity, cell-subtype-specific perturbation of YAP/TAZ signaling, functional assessment by auditory brainstem response and distortion product otoacoustic emission, and long-term evaluation of epithelial stability. This organization is intended to clarify what Hippo–YAP/TAZ signaling has already been shown to do in the inner ear, what remains speculative, and which experiments are required before mechanobiological modulation can be considered a plausible regenerative strategy.

## 1 Introduction

### 1.1 The clinical dilemma and bottlenecks of traditional approaches

Sensorineural hearing loss (SNHL) severely impacts the quality of life for hundreds of millions of people globally (Haile et al., 2021). Its core pathological basis is the irreversible loss or dysfunction of sensory hair cells (HCs) within the organ of Corti, which mature mammals fail to replace efficiently after injury (Xu and Yang, 2021; Qi et al., 2024). Unlike non-mammalian vertebrates, such as birds and zebrafish, which retain robust hair-cell regenerative capacity, the mammalian cochlea shows only limited regenerative potential during early postnatal stages and loses most of this plasticity with maturation (Chai et al., 2012; Bramhall et al., 2014; Cox et al., 2014; Xu and Yang, 2021; Qi et al., 2024). Over the past decades, researchers have attempted to reactivate aspects of regenerative competence in the mammalian cochlea by targeting classical developmental signaling pathways and transcriptional regulators, including Wnt (Wingless-type MMTV integration site family), Notch, Atoh1 (atonal homolog 1), Gfi1 (growth factor independent 1), and Ikzf2 (IKAROS family zinc finger protein 2) (Chai et al., 2012; Shi et al., 2012; Cox et al., 2014; Shu et al., 2019; Sun et al., 2021; Li et al., 2025). Promising progress has been achieved in neonatal mouse models, adult reprogramming paradigms, and organoid systems. However, the generation of mature, correctly patterned, and functionally integrated hair cells in the adult cochlea remains a major unresolved challenge (Shu et al., 2019; Sun et al., 2021; Smith-Cortinez et al., 2023; Qi et al., 2024; Li et al., 2025). This cross-species disparity suggests that mammalian supporting-cell plasticity is restricted by additional barriers that remain incompletely understood.

### 1.2 Why Hippo–YAP/TAZ signaling matters in the inner ear

The Hippo pathway has emerged as a candidate regulator of inner-ear growth, injury responses, and regenerative competence because its downstream effectors, Yes-associated protein (YAP) and transcriptional coactivator with PDZ-binding motif (TAZ), couple tissue architecture, cytoskeletal state, cell density, and extracellular cues to transcriptional programs controlling proliferation and progenitor maintenance (Dupont et al., 2011; Panciera et al., 2017; Moya and Halder, 2019; Driskill and Pan, 2023). In the inner ear, Hippo–YAP/TAZ signaling has been implicated in organ of Corti progenitor self-renewal and sensory epithelial size control (Gnedeva et al., 2020), vestibular supporting-cell regeneration and species-dependent regenerative competence (Rudolf et al., 2020), and experimentally induced supporting-cell proliferation or hair-cell-like outcomes in neonatal and adult cochlear models (Kastan et al., 2021; Lu et al., 2022; Jahanshir et al., 2025). These findings justify a focused synthesis of Hippo-related inner-ear studies before considering broader mechanobiological hypotheses. Hair-cell loss is accompanied by supporting-cell remodeling, epithelial repair responses, changes in apical junctions, and reorganization of the actin cytoskeleton (Cox et al., 2014; Wagner and Shin, 2019; Smith-Cortinez et al., 2023). These processes are essential for epithelial repair, but they may also reduce cellular compliance and limit the morphological changes required for proliferation or fate conversion. Recent evidence from vestibular models, including preclinical and preprint data, suggests that injury-induced remodeling of junctional actin bands may differ between species with high versus low regenerative capacity, raising the possibility that sensory epithelial mechanics influence regenerative potential (Arkenberg et al., 2025; Warchol, 2026).

A detailed evaluation of the biomechanical brake hypothesis is provided after the evidence base for Hippo–YAP/TAZ signaling in the inner ear has been reviewed. Briefly, this hypothesis proposes that injury-associated remodeling of the sensory epithelium may influence Hippo–YAP/TAZ activity and thereby contribute to reduced supporting-cell plasticity in the mature mammalian cochlea (Cox et al., 2014; Wagner and Shin, 2019; Rudolf et al., 2020; Jahanshir et al., 2025). At this stage, however, this model should be regarded as a testable interpretation of available data rather than as an established mechanism.

### 1.3 Core Hippo–YAP/TAZ signaling cascade and mechanosensitive regulation

The Hippo signaling pathway is a highly conserved kinase cascade that integrates diverse intracellular and extracellular cues—including cytoskeletal organization, mechanical force, cell–cell contact, cell polarity, and cell–extracellular matrix interactions—to regulate cell proliferation, differentiation, apoptosis, and tissue homeostasis (Chan et al., 2025). In mammals, the core kinase module consists of the mammalian sterile 20-like kinases 1 and 2 kinases (MST1/2), which associate with the scaffold protein Salvador family WW domain-containing protein 1 (SAV1) to phosphorylate and activate large tumor suppressor kinases 1 and 2 kinases (LATS1/2) in complex with MOB kinase activator 1(MOB1) (Chen et al., 2025; Guo et al., 2025). Activated LATS1/2 then directly phosphorylate the transcriptional coactivators Yes-associated protein (YAP) and transcriptional coactivator with PDZ-binding motif (TAZ) at conserved serine residues (Ser127 in YAP and Ser89 in TAZ). This phosphorylation creates binding sites for 14-3-3 proteins, promoting cytoplasmic sequestration and, in some contexts, ubiquitin-mediated degradation (Chen et al., 2025; Guo et al., 2025). When Hippo signaling is off or attenuated, unphosphorylated YAP/TAZ translocate to the nucleus, where they interact with TEA domain transcription factors (TEAD1-4) to drive transcription of genes involved in proliferation, survival, and progenitor-cell maintenance (Chan et al., 2025; Guo et al., 2025). Beyond this canonical kinase cascade, mitogen-activated protein 4-kinases (MAP4Ks) have been identified as parallel kinases that can activate LATS1/2 independently of MST1/2, adding a layer of regulatory complexity (Chan et al., 2025). Mechanical signals—including extracellular matrix stiffness, actomyosin contractility, and cell density—converge on YAP/TAZ through multiple upstream regulators such as integrin-FAK signaling, Rho family of small GTPases(Rho GTPases), and the neurofibromin 2(NF2)/Moesin-ezrin-radixin-like protein(Merlin) tumor suppressor (Holland et al., 2024; Chen et al., 2025). This mechanosensitivity makes YAP/TAZ attractive candidates for linking physical changes in the tissue microenvironment to cellular regenerative responses.

How mechanical signals reach the core Hippo kinase module has been studied extensively in non-cochlear systems, and three interconnected routes appear most relevant to the cochlear context. Integrin–FAK signaling responds to extracellular matrix stiffness and cytoskeletal tension; FAK autophosphorylation at Tyr397 promotes recruitment of downstream effectors that modulate actin dynamics and, in some settings, suppress LATS activity through Rho GTPase-dependent pathways (Tang et al., 2013; Holland et al., 2024). ROCK-mediated actomyosin contractility regulates cortical tension and cell shape; changes in ROCK activity can alter YAP/TAZ localization indirectly through effects on actin polymerization and nuclear mechanical coupling, as well as through potential cross-talk with the NF2/Merlin tumor suppressor (Panciera et al., 2017; Moya and Halder, 2019). MST/LATS kinases themselves are directly regulated by junctional and polarity signals; proteins such as NF2/Merlin, AMOT, and PALS1 can recruit or activate Hippo components in response to cell–cell contact and epithelial geometry (Moya and Halder, 2019; Chen et al., 2025). These pathways are not strictly linear; FAK and ROCK signals may converge on YAP/TAZ through both LATS-dependent and LATS-independent routes, and the relative contribution of each likely depends on cell type, injury context, and the magnitude of mechanical perturbation.

### 1.4 Hippo–YAP/TAZ signaling as a candidate mechanotransductive hub

In non-cochlear epithelial and stem-cell systems, YAP and TAZ integrate mechanical and biochemical signals with cell-state regulation, and their localization is influenced by extracellular matrix stiffness (Dupont et al., 2011), cell density (Elosegui-Artola et al., 2017), cytoskeletal tension (Panciera et al., 2017), cell polarity (Moya and Halder, 2019), and upstream Hippo kinase activity (Li et al., 2022; Driskill and Pan, 2023). Whether the same logic quantitatively explains adult cochlear supporting-cell restriction remains unresolved. These properties make YAP/TAZ attractive candidates for linking cochlear mechanical remodeling to supporting-cell plasticity, particularly because YAP/TEAD signaling has been implicated in organ of Corti progenitor self-renewal and postnatal supporting-cell proliferation (Gnedeva et al., 2020; Kastan et al., 2021; Lu et al., 2022).

However, direct evidence that injury-induced mechanical remodeling causes YAP/TAZ inactivation in adult mammalian cochlear supporting cells remains limited; most available evidence is derived from developmental, neonatal, *ex vivo*, or non-cochlear mechanobiology models (Gnedeva et al., 2020; Kastan et al., 2021; Lu et al., 2022). A recent study by Jahanshir, Llamas, and colleagues from the Gnedeva laboratory directly addressed this gap by demonstrating that the Hippo pathway and the cell-cycle inhibitor p27Kip1 cooperate to suppress mitotic regeneration in the adult murine organ of Corti (Jahanshir et al., 2025). Using a transgenic model with reduced Cdkn1b expression, the authors showed that pharmacological inhibition of LATS1/2 was sufficient to induce supporting-cell proliferation in the adult cochlea—an effect that was absent when the Hippo pathway was inhibited alone (Jahanshir et al., 2025). This study provides the first direct *in-vivo* evidence from the mature cochlea that relieving Hippo-mediated restriction, in combination with overcoming p27Kip1-mediated cell-cycle blockade, can reactivate proliferative competence in adult auditory supporting cells. A key unresolved question is whether changes in actomyosin tension, focal adhesion signaling, and Hippo kinase activity are sufficient to alter YAP/TAZ localization in defined supporting-cell subtypes *in vivo*. Accordingly, the central premise of this review is deliberately conservative: YAP/TAZ should be considered a candidate mechanotransductive hub and permissive competence regulator, not an established master regulator or sufficient driver of adult cochlear regeneration. The proposed model is summarized in Figure 1.

**FIGURE 1:**
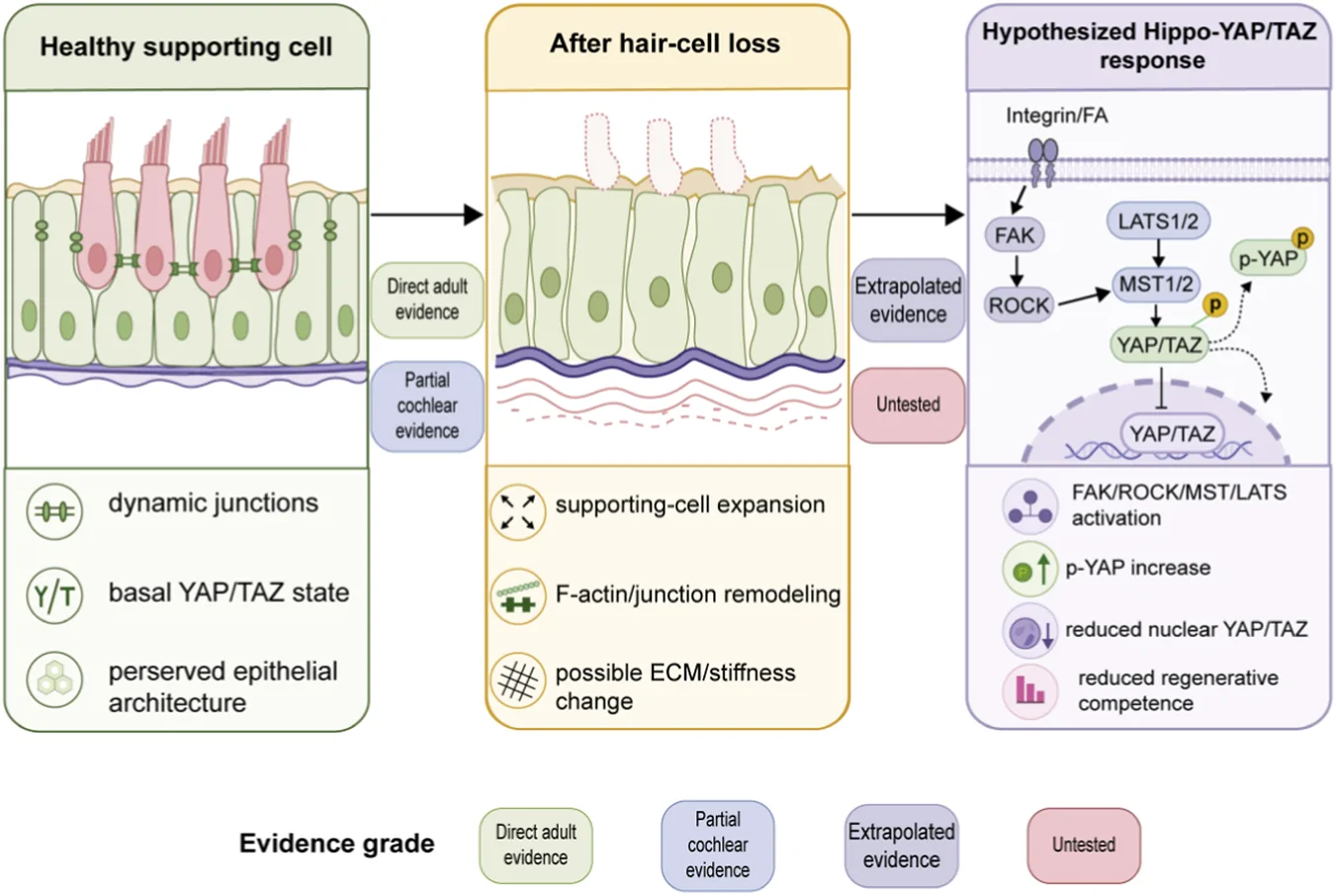
Evidence-based framework linking Hippo–YAP/TAZ signaling to inner-ear development, injury responses, and the biomechanical brake hypothesis. Hair-cell loss is followed by supporting-cell expansion, remodeling of F-actin and epithelial junctions, and possible changes in extracellular matrix organization or local tissue stiffness. These injury-associated changes may influence mechanosensitive signaling pathways, including FAK, ROCK, MST/LATS, and Hippo–YAP/TAZ activity. The model proposes that altered YAP phosphorylation and changes in nuclear YAP/TAZ activity may contribute to reduced regenerative competence in adult cochlear supporting cells, although the direction, timing, and cell-type specificity of these changes remain to be directly established, although the direction, timing, and cell-type specificity of these changes remain to be directly established. Evidence labels indicate the approximate strength of support for each step: direct adult evidence, partial cochlear evidence, extrapolated evidence, or currently untested. The diagram should therefore be interpreted as a testable framework rather than as an established causal pathway.

### 1.5 Scope and structure of this review

In this review, we first provide a structured synthesis of published studies on Hippo–YAP/TAZ signaling in the inner ear, including evidence from inner-ear progenitor cultures, organ of Corti development, vestibular sensory epithelia, cochlear injury models, neonatal regeneration paradigms, and emerging adult *in vivo* studies (Gnedeva et al., 2020; Rudolf et al., 2020; Kastan et al., 2021; Lu et al., 2022; Jahanshir et al., 2025). We then use this evidence base to evaluate the biomechanical brake hypothesis, with particular attention to findings that support, limit, or argue against the model. Finally, we discuss unresolved gaps and propose future studies needed to determine whether Hippo–YAP/TAZ signaling represents a causal, protective, or context-dependent regulator of mammalian inner-ear regeneration.

### 1.6 Scope and evidence grading

Because Hippo–YAP/TAZ signaling has been studied across diverse inner-ear and non-inner-ear systems, the available evidence differs substantially in developmental stage, species, tissue type, injury context, and translational relevance (Gnedeva et al., 2020; Rudolf et al., 2020; Kastan et al., 2021; Lu et al., 2022; Jahanshir et al., 2025). We therefore distinguish among four levels of evidence throughout this review. For each major mechanistic claim, we explicitly separate direct adult *in vivo* evidence from neonatal, *ex vivo*, organoid, and non-cochlear extrapolations, because these categories carry different translational weight. First, direct adult *in vivo* cochlear evidence refers to studies performed in mature mammalian cochleae after acoustic, ototoxic, genetic, or surgical injury. Second, neonatal or juvenile cochlear evidence refers to studies in early postnatal mammalian tissue, where supporting cells retain greater plasticity than in adults. Third, *ex vivo* and organoid evidence includes cochlear explants, inner-ear organoids, and engineered culture systems that allow mechanistic manipulation but incompletely reproduce the adult cochlear niche. Fourth, extrapolated mechanobiological evidence refers to findings from non-cochlear epithelial, stem-cell, fibrotic, or cancer models that inform plausible mechanisms but require validation in the inner ear.

This distinction is critical because a signaling event that promotes proliferation in neonatal supporting cells or non-cochlear epithelia may not necessarily induce functional hair-cell regeneration in the adult organ of Corti. We therefore treat the biomechanical brake hypothesis as a testable interpretation of the Hippo–YAP/TAZ literature rather than as a settled causal pathway. The evidence hierarchy used in this review is intended to distinguish direct adult cochlear evidence from neonatal, *ex vivo*, organoid, vestibular, and non-cochlear mechanobiology evidence. This article is therefore not intended to argue that the biomechanical brake has already been proven. Instead, it first summarizes Hippo–YAP/TAZ signaling studies in the inner ear and then evaluates whether mechanical remodeling and Hippo–YAP/TAZ signaling may constitute a causal, protective, or context-dependent constraint on adult mammalian cochlear regeneration.

To avoid overinterpreting indirect evidence, each mechanistic layer is evaluated using three descriptors: “direct support” for adult *in vivo* cochlear data, “partial support” for neonatal or *ex vivo* cochlear data, and “extrapolated support” for evidence derived primarily from non-cochlear mechanobiology. Claims supported only by extrapolated evidence are treated as hypothesis-generating rather than mechanism-establishing. To provide a clearer overview of the field, Table 1 summarizes published studies that have directly examined Hippo–YAP/TAZ signaling or its major functional readouts in inner-ear models, including the model used, experimental approach, principal conclusion, and major limitation.

**TABLE 1:** Published studies on Hippo–YAP/TAZ signaling in the inner ear.

Study	Model used	Hippo–YAP/TAZ readout or intervention	Main result or conclusion	Major limitation
Xia et al., 2020	Inner-ear progenitor cells in three-dimensional culture	ECM/RhoA/YAP/β-catenin mechanotransductive axis	Mechanical cues regulate progenitor survival, proliferation, and differentiation through RhoA-dependent actin contraction, YAP nuclear accumulation, and β-catenin signaling	*In vitro* progenitor system; does not reproduce the mature organ of Corti niche or auditory function
Gnedeva et al., 2020	Developing mouse organ of Corti and postnatal inner-ear sensory epithelia	Yap/Tead loss- and gain-of-function, including Yap5SA	Yap/Tead regulates progenitor self-renewal, sensory epithelial size, and postnatal cell-cycle re-entry after hair-cell loss	Strong developmental and postnatal evidence, but adult functional hair-cell regeneration remains unresolved
Rudolf et al., 2020	Chicken and mouse vestibular sensory epithelia	YAP activation after damage; LATS-mediated suppression in mice	Damage activates YAP in chicken supporting cells, whereas mammalian LATS activity suppresses YAP responsiveness	Vestibular rather than cochlear model; species differences limit direct extrapolation to adult mammalian cochlea
Borse et al., 2021	Developing and injured mouse and chick utricles	YAP1 localization after sensory epithelial injury	YAP1 localization differs across species and injury contexts, supporting species-specific regulation of regenerative competence	Mainly vestibular localization data; does not establish adult cochlear causality
Kastan et al., 2021	Murine inner-ear sensory epithelia and other postmitotic tissues	Small-molecule LATS inhibition	LATS inhibition reduces Yap phosphorylation, induces supporting-cell proliferation, and can lead to partial expression of sensory hair-cell genes after withdrawal	Supports initial proliferative regeneration but not complete mature or functional cochlear regeneration
Wang et al., 2022	Cochlear hair-cell injury model	YAP activation and inhibition during neomycin-induced damage	YAP activation protects against neomycin-induced hair-cell loss by reducing apoptosis and oxidative stress	Primarily an otoprotection study in aminoglycoside injury; does not address supporting-cell-mediated hair-cell regeneration
Lu et al., 2022	Neonatal mouse organ of Corti	Mst1/2 inhibition, Yap overexpression, and Hippo pathway suppression	Hippo inhibition induces YAP nuclear accumulation in supporting cells and promotes non-mitotic hair-cell generation in neonatal cochlear tissue	Neonatal and largely *ex vivo* context; adult regenerative relevance remains uncertain
Kastan et al., 2022	Mammalian regenerative screening models, including inner-ear-relevant Hippo perturbation	Improved LATS inhibitor development	Provides pharmacological tools for reversible YAP activation and regenerative testing	Tool-development study; inner-ear dosing, specificity, and long-term safety remain to be established
Jahanshir et al., 2025	Adult mouse organ of Corti and retina	LATS inhibition combined with Cdkn1b/p27Kip1 reduction	Hippo inhibition and p27Kip1 reduction cooperate to permit mitotic regeneration in adult sensory tissues	Strong adult evidence for proliferative restriction, but does not prove that injury-induced mechanical remodeling causes Hippo-mediated regenerative failure

## 2 Hippo–YAP/TAZ signaling in the inner ear: evidence from development, injury, and regeneration models

Studies of Hippo–YAP/TAZ signaling in the inner ear can be grouped into five related areas: mechanical regulation of inner-ear progenitors, control of organ of Corti growth during development, vestibular sensory epithelial regeneration, cochlear hair-cell protection after injury, and pharmacological or genetic manipulation of Hippo signaling in neonatal and adult regeneration models (Gnedeva et al., 2020; Rudolf et al., 2020; Xia et al., 2020; Kastan et al., 2021; Lu et al., 2022; Jahanshir et al., 2025). This organization separates what has been directly shown in inner-ear systems from broader mechanobiological principles derived from non-cochlear tissues. It also provides the evidence base needed to evaluate whether Hippo–YAP/TAZ signaling acts primarily as a developmental growth regulator, an injury-response pathway, a permissive regulator of supporting-cell competence, or part of a broader biomechanical restriction in the adult cochlea.

### 2.1 Inner-ear progenitor models and mechanical regulation of YAP activity

Inner-ear progenitor models provide an important entry point for understanding how mechanical cues may influence Hippo–YAP/TAZ signaling in inner-ear lineage cells. In three-dimensional culture, activation of the RhoA–YAP–β-catenin signaling axis has been reported to promote the expansion of inner-ear progenitor cells, suggesting that cytoskeletal organization and extracellular mechanical cues can influence progenitor survival, proliferation, and differentiation (Xia et al., 2020). This evidence supports the biological plausibility that YAP activity is mechanically responsive in inner-ear-related cells. However, these cultures do not reproduce the mature organ of Corti niche, the tonotopic mechanical gradient, or auditory functional constraints, and should therefore be interpreted as reductionist models rather than direct evidence for adult cochlear regeneration.

### 2.2 Yap/Tead signaling in organ of Corti growth and progenitor self-renewal

A central inner-ear study showed that Yap/Tead signaling regulates organ of Corti size by controlling progenitor self-renewal during development (Gnedeva et al., 2020). In that context, YAP activity was linked to progenitor expansion and sensory epithelial growth, placing Hippo–YAP/TAZ signaling upstream of cell-number control in the developing organ of Corti. This developmental evidence is important because it establishes an inner-ear-specific role for Yap/Tead signaling, but it should not be directly equated with adult regenerative competence, where supporting cells face additional barriers such as cell-cycle inhibition, epigenetic restriction, and loss of developmental plasticity (Cox et al., 2014; Smith-Cortinez et al., 2023; Jahanshir et al., 2025).

### 2.3 Vestibular sensory epithelia and species-dependent YAP responsiveness

Vestibular sensory epithelia provide comparative evidence for species-dependent YAP responsiveness during sensory regeneration. In chicken vestibular organs, injury induces YAP activation in supporting cells and is associated with regenerative proliferation, whereas mammalian vestibular epithelia show stronger LATS-mediated suppression of YAP activity and weaker regenerative responses (Rudolf et al., 2020). Additional studies of YAP1 localization in developing and injured utricles further support the view that YAP responsiveness differs across species and injury contexts (Borse et al., 2021). These findings are highly relevant to the biomechanical brake hypothesis because they suggest that mechanically and molecularly regulated YAP activity may contribute to differences in regenerative competence. However, vestibular data should not be treated as direct evidence for adult cochlear regeneration.

### 2.4 Hippo–YAP signaling in cochlear hair-cell injury and otoprotection

Hippo–YAP signaling has also been investigated in cochlear hair-cell injury models. In aminoglycoside-induced cochlear damage, YAP activation has been reported to reduce hair-cell loss, apoptosis, and oxidative stress, suggesting a protective role for YAP in injured cochlear hair cells (Wang et al., 2022). This line of evidence broadens the relevance of Hippo–YAP signaling beyond supporting-cell proliferation. However, otoprotection should be distinguished from regeneration: preservation of existing hair cells does not demonstrate supporting-cell-mediated hair-cell replacement, lineage conversion, or restoration of mature auditory function.

### 2.5 Pharmacological and genetic Hippo manipulation in neonatal cochlear models

Pharmacological and genetic manipulation of Hippo signaling has provided important evidence that YAP/TAZ activity can influence supporting-cell competence in neonatal and postnatal cochlear models. Small-molecule LATS inhibition can reduce YAP phosphorylation and promote supporting-cell proliferation in postmitotic mammalian tissues, including inner-ear sensory epithelia (Kastan et al., 2021). Similarly, loss of Mst1/2 activity or Yap overexpression in neonatal organ of Corti models can induce YAP nuclear accumulation in supporting cells and promote limited hair-cell-like outcomes after injury (Lu et al., 2022). These findings support a role for Hippo signaling in immature cochlear plasticity, but they do not establish that Hippo inhibition alone can restore adult cochlear regeneration (Jahanshir et al., 2025).

### 2.6 Adult cochlear evidence: Hippo signaling, p27Kip1, and proliferative restriction

The strongest adult *in vivo* evidence currently comes from studies showing that Hippo pathway inhibition cooperates with reduction of the cell-cycle inhibitor p27Kip1 to permit supporting-cell proliferation in the mature mouse organ of Corti (Jahanshir et al., 2025). This work is important because it demonstrates that Hippo signaling contributes to adult proliferative restriction, but it also shows that Hippo inhibition alone is insufficient when cell-cycle barriers remain intact. Moreover, this adult evidence does not demonstrate that injury-induced mechanical remodeling is the upstream cause of Hippo-mediated restriction, nor does it establish the generation of mature, synaptically integrated, and functionally effective hair cells. Thus, the adult cochlear evidence supports Hippo signaling as a permissive regulator of proliferative competence rather than as a sufficient driver of complete regeneration.

## 3 From Hippo–YAP/TAZ evidence to the biomechanical brake hypothesis

The evidence summarized above indicates that Hippo–YAP/TAZ signaling can regulate inner-ear progenitor expansion, sensory epithelial growth, vestibular supporting-cell responses, cochlear hair-cell survival, and supporting-cell proliferation under selected experimental conditions. The biomechanical brake hypothesis extends these findings by proposing that injury-associated remodeling of the mature mammalian cochlea may alter mechanical cues that converge on Hippo–YAP/TAZ signaling and thereby restrict supporting-cell plasticity. This hypothesis is attractive because the organ of Corti is a mechanically specialized epithelium in which cellular architecture, junctional organization, cytoskeletal tension, and sensory function are tightly coupled. However, the model remains incompletely proven and must be evaluated against both supporting and limiting evidence. The supportive and limiting evidence for the biomechanical brake hypothesis is summarized in Table 2.

**TABLE 2:** Evidence supporting and limiting the biomechanical brake hypothesis.

Question	Evidence supporting the hypothesis	Evidence limiting or challenging the hypothesis	Balanced interpretation
Can mechanical cues regulate YAP/TAZ-related inner-ear cell behavior?	Inner-ear progenitor and organoid studies suggest that extracellular or cytoskeletal cues can influence YAP localization and progenitor behavior(Xia et al., 2020; Arkenberg et al., 2025)	These systems do not reproduce the mature organ of Corti architecture, stiffness gradients, or auditory function(Roccio and Edge, 2019; Lou et al., 2023)	Supports biological plausibility but not adult cochlear causality
Is YAP activation associated with regenerative competence in sensory epithelia?	Chicken vestibular sensory epithelia show injury-induced YAP activation and supporting-cell proliferation(Rudolf et al., 2020)	Mouse vestibular epithelia show stronger LATS-mediated suppression and weaker regenerative responses(Rudolf et al., 2020; Borse et al., 2021)	Species-dependent YAP responsiveness may contribute to regenerative differences
Can Hippo inhibition promote cochlear supporting-cell plasticity?	Neonatal cochlear models show YAP nuclear accumulation and limited hair-cell-like generation after Hippo pathway suppression(Kastan et al., 2021; Lu et al., 2022)	Neonatal supporting cells retain greater plasticity than adult supporting cells(Cox et al., 2014; Jahanshir et al., 2025)	Supports a role for Hippo signaling in immature cochlear competence
Is Hippo signaling a barrier in the adult cochlea?	Adult organ of Corti studies show that LATS inhibition can cooperate with Cdkn1b reduction to induce supporting-cell proliferation(Jahanshir et al., 2025)	LATS inhibition alone is insufficient, and functional hair-cell regeneration has not been demonstrated(Jahanshir et al., 2025)	Hippo signaling contributes to proliferative restriction but is not the only adult barrier
Does injury-induced mechanical remodeling cause Hippo-mediated restriction?	This is plausible based on cochlear architecture and non-cochlear mechanobiology(Dupont et al., 2011; Panciera et al., 2017; Wagner and Shin, 2019)	Direct adult cochlear evidence linking mechanics, p-LATS/p-YAP, YAP localization, and lineage outcome is lacking	This is the central unproven step of the hypothesis
Could mechanical remodeling be protective?	Supporting-cell expansion and junctional reinforcement help preserve epithelial continuity after hair-cell loss(Cox et al., 2014; Wagner and Shin, 2019)	Excessive relaxation of actomyosin or junctional tension may destabilize the reticular lamina and worsen function	Mechanical remodeling may be both protective and restrictive depending on timing and magnitude
Is YAP/TAZ activation sufficient for regeneration?	YAP activation can support proliferation or competence-state changes(Gnedeva et al., 2020; Kastan et al., 2021; Lu et al., 2022)	Hair-cell subtype identity, bundle maturation, synaptogenesis, and auditory recovery require additional steps(Shu et al., 2019; Sun et al., 2021; Qi et al., 2024)	YAP/TAZ activation is permissive, not sufficient

The references listed in this table are representative examples rather than an exhaustive bibliography for each question. The purpose of the table is to separate evidence that supports the biomechanical brake hypothesis from evidence that limits, challenges, or requires further validation of the model.

Here, we define the biomechanical brake as a testable model in which injury-induced changes in epithelial mechanics, cytoskeletal tension, junctional organization, or extracellular stiffness contribute to reduced supporting-cell plasticity through altered Hippo–YAP/TAZ signaling (Dupont et al., 2011; Panciera et al., 2017; Moya and Halder, 2019; Wagner and Shin, 2019). To support this model, three criteria should be met: mechanical remodeling should be demonstrated in defined adult supporting-cell subtypes; Hippo–YAP/TAZ activity should change in temporal and spatial association with that remodeling; and mechanical or Hippo-axis perturbation should enhance lineage-traced supporting-cell competence without disrupting epithelial integrity (Jahanshir et al., 2025).

## 4 Experimental and therapeutic implications

The evidence reviewed above suggests that Hippo–YAP/TAZ signaling may regulate inner-ear progenitor expansion, supporting-cell competence, and injury responses, but it also indicates that YAP/TAZ activation alone is unlikely to be sufficient for complete adult cochlear regeneration. Experimental strategies should therefore be organized as sequential tests of mechanical permissiveness, Hippo pathway regulation, YAP/TAZ-dependent competence, developmental fate induction, functional maturation, and long-term epithelial safety. These approaches should be regarded primarily as mechanistic perturbations and validation tools rather than as established therapeutic strategies. The proposed sequential experimental framework is summarized in Figure 2.

**FIGURE 2:**
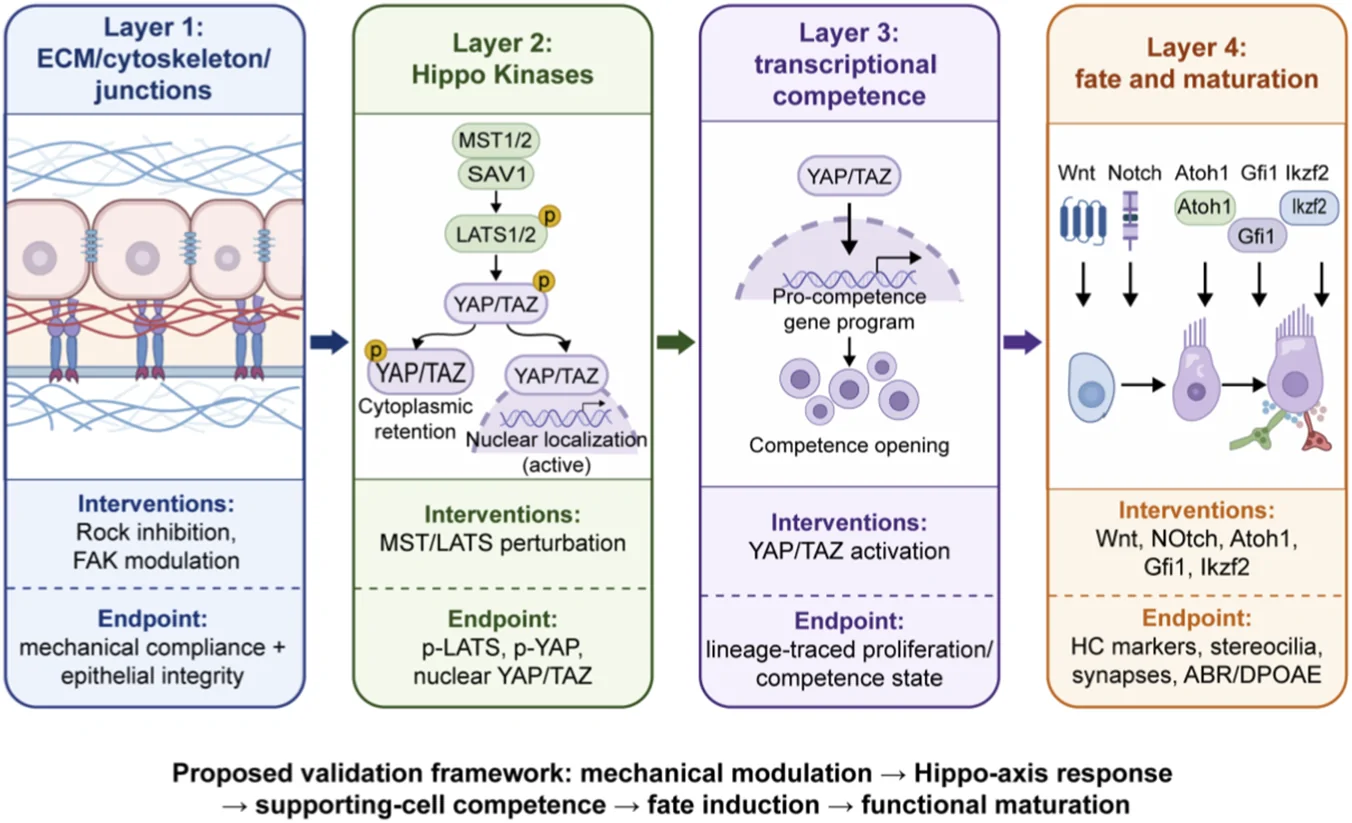
Experimental strategies and validation endpoints for testing Hippo–YAP/TAZ-dependent regenerative competence in the mammalian cochlea. Candidate strategies can be organized from extracellular and cytoskeletal regulation to intracellular Hippo kinase signaling, YAP/TAZ-dependent transcriptional competence, and developmental fate induction. Layer 1 targets extracellular matrix, cytoskeletal, and junctional mechanics through approaches such as ROCK inhibition or FAK modulation, with required endpoints including mechanical compliance and epithelial integrity. Layer 2 targets Hippo kinases through MST/LATS perturbation, requiring assessment of p-LATS, p-YAP, and nuclear YAP/TAZ. Layer 3 evaluates YAP/TAZ activation as a competence-opening event using lineage-traced proliferation and supporting-cell state readouts. Layer 4 integrates developmental regulators such as Wnt, Notch, Atoh1, Gfi1, and Ikzf2, with endpoints including hair-cell markers, stereocilia organization, synaptic integration, and ABR/DPOAE recovery. This framework emphasizes sequential validation from mechanical permissiveness to functional auditory restoration.

### 4.1 Modulating actomyosin contractility through ROCK inhibition

ROCK is a central regulator of actomyosin contractility, myosin light-chain phosphorylation, and cortical tension (Watanabe et al., 2007; Amano et al., 2010). In many epithelial and stem-cell systems, ROCK inhibition can reduce cytoskeletal tension, improve cell survival after dissociation, and alter mechanotransductive signaling, including YAP/TAZ-associated responses in stiffness-sensitive contexts (Watanabe et al., 2007; Amano et al., 2010; Li et al., 2022). In cochlear injury models, ROCK inhibition has been investigated mainly in relation to cellular survival, neurite or synaptic repair, and tissue-protective responses rather than as direct evidence that ROCK inhibition activates YAP-dependent supporting-cell regeneration (Koizumi et al., 2020; Koizumi et al., 2021). Therefore, ROCK inhibitors should be discussed as candidate mechanobiological probes, not as established Hippo–YAP/TAZ activators in adult cochlear supporting cells.

In the context of inner-ear regeneration, ROCK inhibition should be interpreted cautiously. Enhanced survival or improved cellular compliance in culture does not necessarily demonstrate that ROCK inhibition is sufficient to reactivate adult supporting-cell regeneration *in vivo*. A more conservative interpretation is that ROCK inhibition may create a mechanically permissive state that facilitates, but does not by itself specify, regenerative behavior. A decisive test would combine transient local ROCK inhibition with adult supporting-cell lineage tracing, subtype-resolved YAP/TAZ nuclear-to-cytoplasmic quantification, EdU or Ki67 analysis, junctional integrity markers, and subsequent Atoh1/Wnt/Notch-based fate induction to determine whether mechanical relaxation creates a permissive but non-disruptive regenerative window.

### 4.2 Interfering with Integrin–FAK Mechanosensing

Cells sense extracellular matrix composition and stiffness through integrin-containing adhesion complexes, in which focal adhesion kinase (FAK) acts as a major signaling hub connecting adhesion, cytoskeletal tension, and downstream transcriptional responses (Tang et al., 2013; Elosegui-Artola et al., 2017; Lachowski et al., 2018). In non-cochlear systems, FAK can connect extracellular mechanical cues to cytoskeletal remodeling, survival signaling, and Hippo–YAP/TAZ regulation (Tang et al., 2013; Lachowski et al., 2018). This makes FAK a plausible mediator through which a stiffened or remodeled cochlear microenvironment could influence supporting-cell behavior after injury.

Nevertheless, the therapeutic relevance of FAK inhibition in the adult cochlea remains to be established. Although small-molecule FAK inhibitors may reduce mechanosensitive signaling, they may also impair adhesion-dependent survival, epithelial repair, barrier stability, and local inflammatory resolution. Therefore, a positive effect on YAP/TAZ localization alone should not be interpreted as regenerative benefit unless tissue architecture and auditory function are preserved. At the current stage, FAK inhibition should be presented primarily as a mechanistic probe rather than as a therapeutic strategy. The key experiments needed are cell-type-specific measurements of FAK activation after cochlear injury, pharmacological or genetic perturbation of FAK in supporting cells, and simultaneous assessment of YAP/TAZ localization, proliferation, epithelial architecture, and hair-cell fate outcomes.

### 4.3 MST1/2 inhibition: a tool to probe upstream Hippo signaling

MST1/2 are canonical upstream kinases in the Hippo pathway and can contribute to downstream LATS1/2 activation. The small molecule XMU-MP-1 has been used in several non-cochlear tissue-repair contexts to inhibit MST1/2 and enhance YAP-associated repair responses, but its ability to restore regenerative competence in adult cochlear supporting cells remains unproven (Fan et al., 2016; Zhou et al., 2022). In principle, MST1/2 inhibition could help determine whether upstream Hippo signaling limits YAP/TAZ activity in cochlear supporting cells.

However, MST1/2 inhibition may not fully release YAP/TAZ in all contexts because LATS1/2 can also be activated through MST-independent mechanisms, including MAP4K-dependent pathways and signals related to cell polarity or junctional tension (Meng et al., 2016; Moya and Halder, 2019). Therefore, XMU-MP-1 should not be described as a definitive YAP/TAZ activator in the cochlea. The most relevant inner-ear evidence for MST1/2 modulation comes from neonatal organ of Corti models, in which Hippo pathway inhibition or Yap overexpression induces YAP nuclear accumulation in supporting cells and promotes limited hair-cell generation after injury (Lu et al., 2022). These findings support a role for Hippo signaling in neonatal supporting-cell competence, but they should not be interpreted as evidence that MST1/2 inhibition alone can restore adult cochlear regeneration, particularly because adult supporting-cell proliferation appears to require additional release of cell-cycle restriction (Jahanshir et al., 2025). Any claim of regenerative efficacy should require lineage-traced conversion of adult supporting cells into appropriately patterned hair-cell-like cells, acquisition of multiple hair-cell markers beyond Myo7a alone, stereocilia bundle organization, synaptic ribbon formation, and measurable ABR and/or DPOAE improvement.

### 4.4 LATS1/2 inhibition: direct but potentially high-risk YAP/TAZ reactivation

LATS1/2 kinases represent a proximal checkpoint for YAP/TAZ regulation because they directly phosphorylate YAP and TAZ, promoting cytoplasmic retention, 14-3-3 binding, and degradation. Inhibitors of LATS1/2, including TRULI, therefore provide a more direct means of increasing YAP/TAZ activity than upstream MST1/2 inhibition (Kastan et al., 2021; Kastan et al., 2022). This makes LATS inhibition a powerful experimental approach for testing whether YAP/TAZ nuclear exclusion is a limiting barrier in supporting-cell regeneration, although current evidence supports mainly the initial stages of proliferative regeneration rather than complete functional restoration (Kastan et al., 2021).

At the same time, direct LATS inhibition is likely to carry greater biological risk. YAP/TAZ activation can promote proliferation and survival, but uncontrolled or prolonged activation may disrupt epithelial architecture, alter supporting-cell identity, or induce abnormal growth. In the adult organ of Corti, where precise cellular patterning is essential for hearing, even limited epithelial disorganization could compromise function. For this reason, LATS inhibitors should be evaluated using short exposure windows, local delivery, cell-type-specific readouts, and washout or reversibility experiments. The critical question is not whether LATS inhibition can increase nuclear YAP/TAZ, but whether a narrow, reversible therapeutic window exists in which transient YAP/TAZ activation expands supporting-cell competence while preserving epithelial patterning, allowing later cell-cycle exit, hair-cell differentiation, maturation, and auditory function. Importantly, the efficacy of LATS inhibition in the adult cochlea may depend on the concurrent status of cell-cycle inhibitory proteins. Gnedeva and colleagues recently reported that p27Kip1 levels in the organ of Corti are maintained at an unusually high transcriptional level, such that even constitutively active YAP (YAP5SA) fails to overcome this barrier (Jahanshir et al., 2025). Only when Cdkn1b was genetically reduced did LATS inhibition effectively drive supporting-cell proliferation *in vivo* (Jahanshir et al., 2025). This finding suggests that YAP/TAZ reactivation functions as a permissive but insufficient step in the adult cochlea, and that combinatorial strategies targeting both Hippo signaling and cell-cycle checkpoints may be required for regenerative success. This adult *in vivo* evidence is important because it shows that Hippo pathway inhibition can cooperate with reduction of a major cell-cycle barrier to permit supporting-cell proliferation in the mature organ of Corti. However, it does not establish that injury-induced mechanical remodeling is the upstream cause of Hippo-mediated restriction, nor does it demonstrate production of mature, synaptically integrated, and functionally effective hair cells. Thus, this study should be used to support the concept that Hippo signaling contributes to adult proliferative restriction, while the biomechanical origin and regenerative sufficiency of this effect remain unresolved.

### 4.5 AAV-mediated YAP-5SA delivery: a potent experimental test with major safety constraints

Constitutively active YAP variants, such as YAP-5SA, contain serine-to-alanine substitutions at major LATS phosphorylation sites and are therefore less sensitive to canonical LATS-mediated cytoplasmic retention (Zhao et al., 2007; Gnedeva et al., 2020). As an experimental probe, delivery of YAP-5SA to cochlear supporting cells could test whether forced YAP nuclear activity is able to relax selected aspects of postnatal or adult supporting-cell quiescence. Previous studies indicate that pharmacological or genetic activation of YAP can increase supporting-cell proliferation or hair-cell-like outcomes in postnatal or neonatal inner-ear models (Gnedeva et al., 2020; Kastan et al., 2021; Lu et al., 2022). However, these findings should be interpreted as evidence for competence modulation rather than as proof of complete, functional hair-cell regeneration, particularly in the adult cochlea (Jahanshir et al., 2025). This approach should not be framed as a near-term therapy because delivery specificity, reversibility, dosage control, and long-term safety remain major barriers (Landegger et al., 2017; Suzuki et al., 2017; Omichi et al., 2019).

This strategy is best treated as a mechanistic perturbation rather than a therapeutic candidate until lineage, architecture, and functional endpoints are met. YAP-5SA may promote cell-cycle re-entry, but lineage-expanded supporting cells would still need to undergo timely YAP downregulation or contextual rewiring before acquiring a stable hair-cell fate, exiting the cell cycle, organizing stereocilia, forming synaptic contacts, and contributing to auditory recovery. Future studies should therefore use supporting-cell-specific promoters, inducible or self-limiting expression systems, lineage tracing, long-term histological assessment, and functional auditory testing.

### 4.6 Genetic reduction of Hippo pathway inhibitors

An alternative to overexpressing activated YAP is to reduce inhibitory components of the Hippo pathway, such as LATS1/2, NF2, or other upstream regulators, using RNA interference or genome-editing approaches (Moya and Halder, 2019; Kastan et al., 2021; Driskill and Pan, 2023). These strategies could, in principle, provide more pathway-specific evidence for the role of endogenous Hippo signaling in supporting-cell restriction.

However, durable genetic suppression of tumor-suppressive Hippo components raises substantial safety concerns, especially in a mechanically specialized epithelium where even small deviations in cell number, polarity, or supporting-cell identity may degrade cochlear mechanics rather than improve hearing. In the cochlea, where regeneration must occur without compromising epithelial mechanics and sensory patterning, permanent or poorly controlled pathway inhibition may be undesirable. Therefore, future work should prioritize transient, spatially restricted, and cell-type-specific perturbations. Essential endpoints should include not only YAP/TAZ localization and proliferation, but also supporting-cell survival, epithelial integrity, hair-cell differentiation, synapse formation, auditory function, and long-term absence of dysplasia or ectopic growth.

### 4.7 Sequential coupling of YAP/TAZ-mediated competence with developmental fate induction

Reactivating YAP/TAZ may increase supporting-cell plasticity or proliferative competence, but it is unlikely to be sufficient by itself to generate mature and functional hair cells. Hair-cell regeneration requires at least three separable phases: first, generation or reactivation of a competent supporting-cell state; second, lineage specification toward inner or outer hair-cell identity; and third, structural maturation, synaptic integration, and functional incorporation into the cochlear epithelium (Shu et al., 2019; Sun et al., 2021; Qi et al., 2024; Li et al., 2025). YAP/TAZ signaling may primarily contribute to the first process by creating a permissive state for proliferation, survival, or progenitor-like behavior. Developmental pathways and transcriptional regulators such as Wnt, Notch, Atoh1, Gfi1, Ikzf2, FGF, and retinoic acid signaling are likely to be required to guide lineage specification, differentiation, and maturation (Kopan and Ilagan, 2009; Chai et al., 2012; Shi et al., 2012; Shu et al., 2019; Sun et al., 2021; Li et al., 2025).

### 4.8 YAP/TAZ and Wnt/β-catenin crosstalk: expanding competence rather than ensuring fate

Canonical Wnt/β-catenin signaling supports progenitor expansion during inner-ear development and has been widely investigated as a strategy to enhance supporting-cell plasticity (Chai et al., 2012; Shi et al., 2012; Li et al., 2025). Because YAP/TAZ and β-catenin interact in a context-dependent manner, combined Hippo and Wnt modulation may expand or stabilize a progenitor-like state in some cochlear contexts. Whether this state remains competent for ordered hair-cell differentiation in the adult cochlea must be tested rather than assumed (Panciera et al., 2017; Moya and Halder, 2019; Driskill and Pan, 2023).

However, Wnt activation should not be assumed to guarantee hair-cell fate. Excessive or prolonged Wnt signaling may maintain progenitor-like states, alter tissue patterning, or fail to drive terminal differentiation without additional transcriptional cues. A more plausible strategy is temporally controlled activation, in which YAP/TAZ and Wnt signaling are used transiently to expand or prime competent supporting cells, followed by a separate differentiation phase. Future studies should define the optimal timing and dose of Wnt activation relative to YAP/TAZ modulation, and should evaluate whether the resulting cells progress beyond proliferation to mature hair-cell identity and function.

### 4.9 YAP/TAZ and notch signaling: the importance of timing

Notch signaling maintains supporting-cell identity and mediates lateral inhibition during inner-ear development, although recent evidence suggests that specific Notch ligands may also preserve progenitor-like properties in cochlear supporting cells (Kopan and Ilagan, 2009; Sun et al., 2021). Pharmacological Notch inhibition can induce aspects of hair-cell fate in some neonatal or damaged cochlear contexts, but its effects in the mature mammalian cochlea are limited and highly context dependent. Combining YAP/TAZ activation with Notch modulation is therefore conceptually attractive, but the timing and direction of Notch manipulation are likely to be critical (Kopan and Ilagan, 2009; Shu et al., 2019; Lu et al., 2022).

Premature Notch inhibition could deplete the supporting-cell pool or induce incomplete transdifferentiation before sufficient progenitor expansion has occurred. Conversely, prolonged YAP/TAZ activation may maintain proliferation or interfere with terminal differentiation. A testable sequential strategy would involve a short competence-opening phase using YAP/TAZ and/or Wnt activation, a defined withdrawal phase to permit cell-cycle exit and prevent epithelial overgrowth, and a subsequent fate-induction phase using Notch modulation and hair-cell transcriptional regulators such as Atoh1, Gfi1, or Ikzf2. This sequence should be tested using lineage tracing, single-cell transcriptomics, hair-cell maturation markers, stereocilia morphology, synaptic markers, and functional assays.

### 4.10 Comparative considerations across intervention layers

The intervention layers described above differ considerably in mechanistic specificity, translational readiness, and risk. ROCK and FAK inhibition target upstream mechanical signaling and may have broad effects on cell survival, adhesion, and epithelial integrity; they are useful for probing the mechanical component of the brake but are less suitable for therapeutic development until their effects on cochlear architecture are better characterized. MST1/2 inhibition acts more proximally to Hippo signaling but may be insufficient when LATS remains active through parallel pathways. LATS1/2 inhibition provides a direct means of YAP/TAZ release but carries the highest risk of uncontrolled proliferation or epithelial disruption, and its efficacy may depend on concurrent cell-cycle inhibitory status. Genetic strategies such as YAP-5SA offer the greatest potency and experimental specificity but face substantial delivery and safety barriers. Developmental pathway modulators do not directly target the mechanical or Hippo axis but are likely necessary for fate specification once proliferative competence has been achieved. Temporally ordered combinations—rather than single-agent strategies—are therefore likely to be most informative for testing the sequential nature of the brake hypothesis, though they introduce additional complexity in dosing and sequencing that must be determined empirically.

## 5 Gaps and future studies

### 5.1 Defining regeneration beyond YAP/TAZ activation

A central limitation of current YAP/TAZ-centered strategies is that nuclear YAP/TAZ activity and supporting-cell proliferation are early and permissive events, not definitive evidence of hair-cell regeneration. Functional regeneration requires a sequence of distinct biological events: specification of hair-cell identity, acquisition of inner or outer hair-cell subtype features, formation of organized stereocilia bundles, establishment of ribbon synapses with spiral ganglion neurons, survival within the mature cochlear environment, and measurable improvement in auditory function (Shu et al., 2019; Wagner and Shin, 2019; Sun et al., 2021; Qi et al., 2024; Li et al., 2025). Therefore, proliferation or Myo7a expression alone should not be treated as sufficient evidence of regeneration. A staged endpoint hierarchy is needed. Early endpoints should include adult supporting-cell lineage tracing, EdU or Ki67 incorporation, and competence-state markers. Intermediate endpoints should include acquisition of multiple hair-cell markers beyond Myo7a, assignment of inner or outer hair-cell subtype identity, and formation of organized stereocilia bundles. Late endpoints should include mechanotransduction currents or equivalent physiological readouts, ribbon synapse formation, ABR and DPOAE improvement, and long-term exclusion of epithelial disorganization, ectopic proliferation, or dysplasia.

### 5.2 Resolving supporting-cell subtype and tonotopic heterogeneity

Supporting cells are not a uniform population, and their regenerative potential differs according to subtype, cochlear region, tonotopic position, age, and injury context. Therefore, conclusions drawn from one supporting-cell subtype, one cochlear turn, or one neonatal injury model should not be generalized to the adult organ of Corti without subtype-resolved validation (Bramhall et al., 2014; Cox et al., 2014; Smith-Cortinez et al., 2023). Inner pillar cells, inner phalangeal cells, Deiters’ cells, Hensen’s cells, Claudius cells, and other epithelial populations differ in developmental origin, gene-expression profile, mechanical environment, and regenerative competence. Moreover, supporting-cell responses vary along the tonotopic axis and change dramatically with age. A mechanobiological model of regeneration must therefore account for cell-type-specific and region-specific differences in YAP/TAZ activity, Wnt responsiveness, Notch signaling, chromatin accessibility, and cell-cycle control. Future studies should combine lineage tracing with single-cell or spatial transcriptomics to identify which supporting-cell populations are most responsive to mechanical or Hippo pathway perturbation.

### 5.3 Mechanical remodeling may be protective as well as restrictive

Mechanical remodeling after hair-cell loss should not be interpreted solely as pathological. Supporting-cell expansion, actin reinforcement, and junctional remodeling help preserve epithelial continuity and protect the cochlear partition after injury. The same processes, however, may also reduce cellular compliance and limit the morphological changes required for proliferation or fate conversion. Future studies should therefore treat mechanical remodeling as a potentially dual-function response and should evaluate whether ROCK, FAK, or Hippo-axis perturbation improves supporting-cell competence without destabilizing the reticular lamina, epithelial polarity, or cochlear micromechanics. The recent identification of p27Kip1 as a Hippo-cooperating barrier in the adult organ of Corti has important implications for the biomechanical brake hypothesis (Jahanshir et al., 2025). If mechanical remodeling acts upstream of both Hippo-YAP/TAZ inactivation and p27Kip1 maintenance, then strategies targeting mechanical tension alone may be insufficient unless they also address cell-cycle inhibitory mechanisms. Conversely, if mechanical changes and p27Kip1 expression are independently regulated, the biomechanical brake may represent only one component of a multi-layered restriction—a possibility that aligns with the emerging view that Hippo signaling and cell-cycle inhibitors function synergistically to enforce quiescence in adult mammalian sensory epithelia (Jahanshir et al., 2025).

### 5.4 When mechanical relaxation May Be detrimental

An important counter-hypothesis is that the same mechanical remodeling proposed to restrict regeneration may also be required to preserve cochlear function after injury. ROCK, FAK, or LATS inhibition could increase YAP/TAZ activity or cellular compliance, yet still worsen outcomes if it disrupts junctional sealing, supporting-cell polarity, reticular lamina integrity, or organ of Corti micromechanics. Therefore, future studies should treat epithelial stability and auditory function as co-primary endpoints rather than secondary safety readouts. A mechanobiological intervention should be considered beneficial only if it enhances supporting-cell competence without compromising tissue architecture or cochlear mechanics.

### 5.5 Safety, reversibility, and long-term epithelial stability

Because the Hippo–YAP/TAZ pathway regulates proliferation, survival, stem-cell activity, organ size, and tumorigenesis-associated processes, its manipulation requires strict safety controls (Panciera et al., 2017; Moya and Halder, 2019; Driskill and Pan, 2023). The most clinically plausible strategies will likely require local delivery, transient and titratable activation, supporting-cell-subtype specificity, reversibility, and built-in termination mechanisms. In the absence of these features, Hippo–YAP/TAZ activation should be regarded as a high-risk experimental manipulation rather than a translational therapy. Long-term YAP/TAZ activation, especially through constitutively active YAP variants or durable suppression of LATS1/2, may carry risks of abnormal proliferation, loss of supporting-cell identity, or epithelial disorganization. Preclinical studies should therefore include extended follow-up, dose-response analysis, off-target assessment, and histological evaluation of the entire cochlear epithelium.

The next stage of research should move beyond broad pathway activation and toward decisive, spatially resolved experiments that can validate or falsify the biomechanical brake hypothesis. The key challenge is to distinguish among three possibilities: mechanical remodeling may act as a causal barrier to regeneration, as a protective secondary repair response, or as a context-dependent process with dual effects depending on timing, magnitude, supporting-cell subtype, and cochlear region. Addressing this question will require models that preserve the architecture of the mature cochlea, tools that measure mechanical properties at cellular resolution, and perturbations that can be restricted to defined supporting-cell subtypes.

### 5.6 Engineering the mechanical niche in organoids and cochlear explants

Conventional two-dimensional culture and standard Matrigel-based organoids provide valuable experimental access but do not fully reproduce the stiffness gradients, epithelial architecture, and mechanical constraints of the mature organ of Corti (Roccio and Edge, 2019; Lou et al., 2023; Van der Valk et al., 2026). The development of tunable hydrogel systems with defined stiffness and viscoelastic properties has enabled systematic interrogation of how mechanical cues influence YAP/TAZ localization and stem-cell behavior, providing a reductionist platform for studying mechanotransduction in inner-ear cell types (Rudolf et al., 2020; Arkenberg et al., 2025). Engineered biomaterials with tunable stiffness and viscoelasticity, such as PEG-based or gelatin methacryloyl hydrogels, may provide reductionist platforms for testing how defined mechanical environments influence YAP/TAZ localization, progenitor-state maintenance, supporting-cell-like behavior, and hair-cell differentiation (Tibbitt and Anseth, 2009; Li et al., 2022; Arkenberg et al., 2025). These platforms should be interpreted as hypothesis-generating systems rather than substitutes for the mature cochlear niche because they incompletely reproduce the geometry, stiffness gradients, epithelial tension, immune context, and micromechanical demands of the adult organ of Corti (Roccio and Edge, 2019; Lou et al., 2023). For example, supporting cells or inner-ear organoids could be cultured across defined stiffness ranges while monitoring F-actin organization, p-FAK, p-LATS, p-YAP, YAP/TAZ nuclear localization, proliferation, and hair-cell differentiation. Such experiments would help determine whether mechanical softening is sufficient to enhance regenerative competence or whether it must be combined with developmental pathway modulation.

### 5.7 Mapping the cochlear mechanome

A priority for future work is to construct a spatial cochlear mechanome that links local mechanical properties with Hippo pathway activity and supporting-cell state after injury (Dupont et al., 2011; Elosegui-Artola et al., 2017; Li et al., 2022). In adult cochlear injury models, stiffness-mapping approaches such as atomic force microscopy or related mechanical imaging methods should be combined with immunostaining for F-actin, junctional proteins, p-FAK, p-LATS, p-YAP, and nuclear YAP/TAZ. These measurements should be integrated with lineage tracing, single-cell or spatial transcriptomics, cell-cycle markers, and hair-cell fate markers in the same experimental system. Such experiments would test whether mechanically stressed regions or supporting-cell subtypes show Hippo pathway changes that predict reduced or enhanced regenerative competence (Jahanshir et al., 2025).

A spatial “cochlear mechanome” map would provide a direct test of the model by asking whether regions or supporting-cell subtypes with higher mechanical stress show stronger p-FAK/p-LATS/p-YAP signaling, reduced nuclear YAP/TAZ, lower chromatin accessibility at regenerative loci, and weaker lineage-traced regenerative responses. It would also identify supporting-cell subtypes or cochlear regions that are most likely to respond to mechanobiological intervention. The major experimental strategies, mechanistic rationales, limitations, and validation endpoints for testing Hippo–YAP/TAZ-dependent regenerative competence are summarized in Table 3.

**TABLE 3:** Experimental strategies and validation endpoints for testing Hippo–YAP/TAZ-dependent regenerative competence.

Experimental layer	Candidate strategy	Mechanistic rationale	Required validation endpoints	Main risk or limitation
Mechanical niche	ROCK inhibition, FAK modulation, ECM stiffness tuning	Tests whether cytoskeletal tension or extracellular mechanics influences Hippo–YAP/TAZ activity	Tissue stiffness, F-actin organization, junctional integrity, p-FAK, p-YAP, nuclear YAP/TAZ, and epithelial stability	Mechanical relaxation may destabilize reticular lamina integrity or cochlear micromechanics
Hippo kinase modulation	MST1/2 or LATS1/2 perturbation	Tests whether Hippo kinase activity limits YAP/TAZ-dependent supporting-cell competence	p-MST, p-LATS, p-YAP, nuclear YAP/TAZ, supporting-cell proliferation, and lineage tracing	LATS inhibition may cause uncontrolled proliferation or epithelial disorganization
YAP/TAZ transcriptional activation	YAP5SA, transient YAP activation, or TEAD-dependent readouts	Tests whether YAP/TAZ activation opens a competence state in supporting cells	Supporting-cell lineage tracing, EdU or Ki67 incorporation, competence-state markers, and withdrawal or reversibility assays	Proliferation may not lead to hair-cell fate conversion or functional maturation
Developmental fate induction	Sequential modulation of Wnt, Notch, Atoh1, Gfi1, Ikzf2, or related developmental regulators	Tests whether competence opening can be coupled to hair-cell specification	Multiple hair-cell markers, inner or outer hair-cell subtype identity, stereocilia bundles, and synaptic ribbon formation	Incorrect timing may cause incomplete differentiation, supporting-cell depletion, or epithelial patterning defects
Functional validation	Auditory and structural outcome testing	Determines whether regenerated or induced cells contribute to cochlear function	Mechanotransduction currents or equivalent physiological readouts, ABR, DPOAE, synapse analysis, and long-term histology	Functional recovery may fail despite molecular or histological improvement
Safety and reversibility	Local delivery, transient activation, inducible systems, washout experiments, and long-term follow-up	Tests translational feasibility and tissue stability	Dose-response analysis, off-target assessment, epithelial polarity, absence of ectopic proliferation, and absence of dysplasia	Durable Hippo suppression or uncontrolled YAP/TAZ activation carries safety risks

These strategies are proposed as experimental validation steps rather than established therapeutic approaches. Each intervention should be interpreted in relation to supporting-cell subtype, cochlear region, developmental stage, injury model, and long-term epithelial safety. YAP, Yes-associated protein; TAZ, transcriptional coactivator with PDZ-binding motif; FAK, focal adhesion kinase; ECM, extracellular matrix; MST, mammalian sterile 20-like kinase; LATS, large tumor suppressor kinase; TEAD, TEA, domain transcription factor; ABR, auditory brainstem response; DPOAE, distortion product otoacoustic emission.

## 6 Conclusion

Published studies indicate that Hippo–YAP/TAZ signaling is involved in multiple inner-ear contexts, including inner-ear progenitor expansion, organ of Corti growth, vestibular supporting-cell responses, cochlear hair-cell protection, and experimentally induced supporting-cell proliferation. These findings establish Hippo–YAP/TAZ signaling as an important regulatory axis in inner-ear biology, but they do not yet demonstrate that YAP/TAZ activation alone is sufficient for adult mammalian cochlear regeneration. The mature cochlea remains constrained by multiple barriers, including cell-cycle inhibition, epigenetic restriction, loss of developmental competence, supporting-cell heterogeneity, and the need to preserve epithelial mechanics.

The biomechanical brake hypothesis should therefore be viewed as a secondary, evidence-graded interpretation of the Hippo–YAP/TAZ literature rather than as a settled mechanism. Its value lies in generating testable predictions: adult cochlear injury should produce defined mechanical changes in supporting-cell subtypes; these changes should correlate with Hippo pathway activity and YAP/TAZ localization; and targeted perturbation should improve lineage-traced supporting-cell competence without compromising epithelial integrity. At present, the strongest evidence supports YAP/TAZ as a permissive regulator of proliferation or competence, whereas hair-cell fate specification, maturation, synaptic integration, and auditory recovery require additional regulatory steps.

Future progress will depend on evidence that directly links mechanical remodeling to Hippo–YAP/TAZ activity in adult cochlear supporting cells, and on interventions that can safely and reversibly modulate this axis *in vivo*. Future studies should combine adult supporting-cell lineage tracing, spatial mechanome mapping, subtype-resolved Hippo pathway readouts, temporally controlled YAP/TAZ perturbation, developmental fate induction, and functional auditory assessment. These experiments will be necessary to determine whether mechanical remodeling is a causal barrier, a protective repair response, or a context-dependent modulator of Hippo–YAP/TAZ-regulated regenerative competence in the mammalian cochlea.
